# Analysing calcium signalling of cells under high shear flows using discontinuous dielectrophoresis

**DOI:** 10.1038/srep11973

**Published:** 2015-07-23

**Authors:** Rebecca Soffe, Sara Baratchi, Shi-Yang Tang, Mahyar Nasabi, Peter McIntyre, Arnan Mitchell, Khashayar Khoshmanesh

**Affiliations:** 1 School of Electrical and Computer Engineering, RMIT University, Melbourne, Australia; 2 Health Innovations Research Institute and School of Medical Sciences, RMIT University, Melbourne, Australia

## Abstract

Immobilisation of cells is an important feature of many cellular assays, as it enables the physical/chemical stimulation of cells; whilst, monitoring cellular processes using microscopic techniques. Current approaches for immobilising cells, however, are hampered by time-consuming processes, the need for specific antibodies or coatings, and adverse effects on cell integrity. Here, we present a dielectrophoresis-based approach for the robust immobilisation of cells, and analysis of their responses under high shear flows. This approach is quick and label-free, and more importantly, minimises the adverse effects of electric field on the cell integrity, by activating the field for a short duration of 120 s, just long enough to immobilise the cells, after which cell culture media (such as HEPES) is flushed through the platform. In optimal conditions, at least 90% of the cells remained stably immobilised, when exposed to a shear stress of 63 dyn/cm^2^. This approach was used to examine the shear-induced calcium signalling of HEK-293 cells expressing a mechanosensitive ion channel, transient receptor potential vaniloid type 4 (TRPV4), when exposed to the full physiological range of shear stress.

The ability to stably immobilise cells is an important feature in cellular assays, as it enables the physical/chemical stimulation of cells and monitoring of cellular processes using a variety of microscopic techniques^1^. Classically, the immobilisation of non-adherent cells is acieved by surface modification^2^, which can be accomplished in different ways: such as coating the substrate surface with biomimetic peptides like poly L-lysine or poly ornithine^3^,^4^; cell adhesive proteins like laminin or fibronectin^5^; or patterning a suitable ligand onto the substrate which allows cells to attach, spread and migrate along the surface^6^,^7^.

Important drawbacks of such surface modification approaches are the protein adsorption into the substrate, and the interaction between the cell-substrate may be influenced by different parameters such as surface free energy, charge, roughness, and thickness of modifying layer. Consequently, these surface modifications are often unstable and uneven, and can lead to cellular rearrangement when exposed to a high magnitude of mechanical forces^5^. Furthermore, any surface modification can affect the biology of cells and consequently change cellular responses to the experimental conditions. As such, this approach is not ideal for immobilisation of non-adherent cells, especially when high levels of mechanical stress such as flow-induced shear is required.

Microfluidic systems are widely considered, as enabling technologies in cellular biology research^8^,^9^,^10^. Microfluidic platforms offer reduced sample and reagent volumes, sample diversity, short reaction times, enhanced sensitivity, and the capacity for multiplexing and automation^1^,^8^,^11^. Moreover, microfluidic systems enable the quick and controllable immobilisation of cells using a variety of mechanisms, including hydrodynamics^12^, optical tweezing^13^, acoustophoresis^14^, magnetophoresis^15^, and dielectrophoresis^16^,^17^.

The use of hydrodynamic filters can lead to clogging of the microfluidic channel by trapped cells or debris^18^,^19^. Moreover, the performance of such filters depends on the size and deformability of the cells, such that the filters may need to be redesigned for different cell types^12^,^19^. In addition, the trapping of cells between structures can potentially limit the amount of shear stress, which can be applied onto the cells^18^,^20^. Although the use of hydrogels has enabled cells to be immobilised into three dimensional structures, this process is limited to the use of low flow rates, which are not suitable for the investigation of shear-induced stress^21^,^22^.

Alternatively, “Optical tweezers” rely on sophisticated optical components to produce the desired optical patterns, in particular for producing multi-beam interference patterns for multiple immobilised cells clusters^13^,^23^. In addition, the exposure of cells to highly focused laser beams can damage them or alter the functionality of cellular proteins^24^. Acoustophoresis enables the label-free and non-invasive manipulation of both single and multiple cells^14^,^25^. However, the precise control on the vertical location of cells within the microfluidic channel can be challenging, and the cells focused at the same pressure node can be stacked on top of each other. Magnetic tweezers, on the other hand, require the labelling of cells with immuno-magnetic tags^15^.

Dielectrophoresis, the induced motion of polarisable particles such as cells under the influence of non-uniform electric fields, enables the label-free, selective and quick immobilisation of cells in microfluidic systems^16^,^17^,^26^,^27^,^28^. Despite these advantages, the long-term exposure of cells to strong electric fields may affect the viability, and functioning of cells^17^. The temperature rise of the medium due to Joule heating effect is another factor that can damage cells^29^. Moreover, the electrical conductivity of the buffer should be reduced to enable the immobilisation of cells, which can damage them in long-term experiments^30^. The immobilised cells can also be exposed to unwanted chemical reactions such as electrolysis, which might happen over the surface of microelectrodes^29^.

Several approaches have been implemented to address these limitations. One such approach is reducing the amount of time that cells are immobilised between the microelectrodes, which is suggested to reduce the negative impacts of strong electric fields, and also temperature rise on cells. In this method, the microelectrodes are switched on/off periodically to enable the quick trap/release of cells. Using this method, Hawkins *et al.*^31^ limited the immobilisation time of *Mycobacterium smegmatis* to three seconds, just long enough to measure mycobacterial membrane properties, after which the cells were released. Similarly, Zhang *et al.*^32^ limited the immobilisation time of yeast cells to 30 ms, just long enough to obtain the Raman signature of the cells, after which cells were released to be sorted based on their Raman spectra. Evidently, this solution does not allow for long-term analysis of cellular responses.

An alternative approach for maintaining cell viability and functioning, involves reducing the amount of time in which immobilised cells are exposed to strong electric fields. For example, in the work presented by Khoshmanesh *et al.*^30^ the microelectrodes were energised with a voltage of 12 V_pk-pk_ for 10 min to immobilise the suspended leukemic U937 cells. After which the voltage was reduced to 8 V_pk-pk_, just high enough to produce electrothermal vortices within the microfluidic chamber, to enable the constant perfusion of cells with apoptosis inducer drugs over a duration of four hours. Despite some advantages, this system does not allow for long-term experiments, as the cells need to be kept in a low electrical conductivity (LEC) buffer.

Alternatively, Yang *et al.*^33^ pre-coated the microelectrodes with Anti-*Salmonella* antibodies. The electric field was applied for a period of 15 to 30 min, to push the *Salmonella* bacterial cells towards the antibody-coated microelectrodes. This not only enhanced the immune-capture efficiency of cells, but also enabled the stable adhesion of cells once the electric field was switched off. Despite these advantages, the pre-coating of microelectrodes with antibodies complicates the operational process and is specific. Instead, in the work presented by Sebastian *et al.*^34^ the electric field was only activated for 10 to 15 minutes to create aggregations of Jukart cells over the microelectrodes; after which the electric field was switched off and the LEC buffer was replaced with growth media. The capability of this technique was demonstrated for creating three-dimensional aggregations of cells; however, the aggregated cells were scattered and slightly relocated after a period of one hour under a low shear stress of 0.012 dyn/cm^2^.

In this paper, we demonstrate a dielectrophoresis-based approach for stable immobilisation of cells and analysis of cellular response under high shear flows. This approach is quick and label-free and does not rely on surface modification. Additionally, it minimises the adverse effects of strong electric fields or low electrical conductivity buffers, on the viability and functioning of cells. This is achieved by activating the field for a short duration of 120 s, just long enough to immobilise the cells, after which cell culture media such as HEPES is flushed through the microfluidic system.

For cell model system, we used HEK-293 cells stably expressing TRPV4 channels, in suspension. TRPV4 is a mechanosensitive calcium permeable ion channels belong to the TRP (transient receptor potential) family of ion channels, which is known to play a key role in controlling vascular homeostasis and tone^35^,^36^,^37^. HEK-293 cells are an excellent cell line to transfect and study TRP channels, as they are easy to work with, and express a very limited range of TRP channels which does not include TRPV4. Such a cell line has been used by us and others to study the characteristics of TRPV4^38^,^39^. However, one common problem with HEK-293 cells (and many other cell lines) is that they do not adhere well to the glass or plastic substrates, which precludes the study of their response to high magnitudes of shear stress. Hemodynamic forces in the venus and arterial systems are in the range of 10 to 60 dyn/cm^2^ 40. However, for studying the effect of shear stress on TRPV4-expressing cells, the shear stress is limited to 20 dyn/cm^2^ or less, largely due to the challenges associated with cells detaching at high magnitudes of shear stress^36^,^38^. Therefore, the response of the TRPV4 channel to the full physiological range of shear stress has not yet been studied. In the present study, up to 90% of the cells remained stably immobilised, when applying a shear stress of 63 dyn/cm^2^. This enabled us to examine the shear-induced calcium signalling of HEK-293 cells, stably expressing TRPV4 at the full physiological range of shear stress and; additionally, these results were compared to results obtained with non-transfected cells.

## Results

### Principles of dielectrophoretic platform

**Figure 1:**
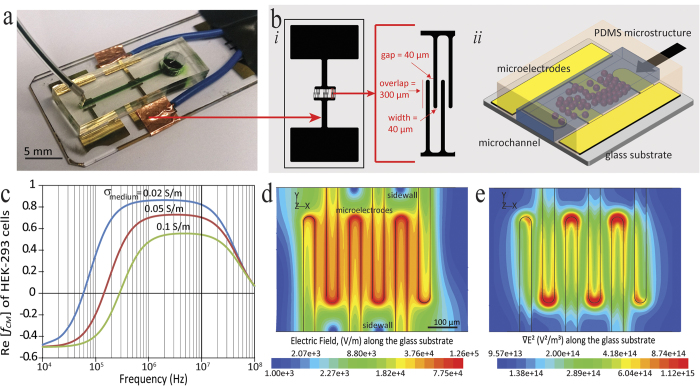
Microfluidic platform used for studying robust immobilisation of cells. (**a**) The actual microfluidic platform. (**b**) Plan view of inter-digital microelectrode design with dimensions of microelectrodes. (**c**) Frequency variation of the real part of the Clausius-Mossotti factor, indicating the DEP response of HEK-293 cells at different frequencies. (**d**) Contours of electric field at the surface of glass substrate, obtained by numerical simulations at an operating voltage of 5 V_pk-pk_. (**e**) Contours of the gradient of electric field square at the surface of glass substrate, obtained by numerical simulations at an operating voltage of 5 V_pk-pk_.

The dielectrophoresis-based microfluidic platform used for the robust immobilisation of cells is displayed as Fig. 1a. This platform utilised inter-digital microelectrodes to create a uniform layer of immobilised cells over the surface of glass substrate. The gap between the microelectrodes and also the width of the microelectrodes were set to 40 μm due to the microfabrication limitations; whilst, the overlap length of opposite microelectrodes was set to 300 μm (Fig. 1b). A polydimethylsiloxane (PDMS) microfluidic channel with cross-sectional dimensions of 500 × 80 μm (W × H), was integrated onto the glass substrate patterned with microelectrodes to facilitate the passage of cells; as schematically shown in Fig. 1b. A reservoir with a diameter of 5 mm was punched at the inlet of the channel, enabling the cell suspension to be applied to the device, whilst, the flow was withdrawn from the outlet.

The time-averaged DEP force applied on cells can be expressed as below^29^:


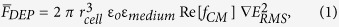


where, *r*_*cell*_ is the radius of the cell, *ε*_*o*_ is the permittivity of the vacuum which equals to 8.85 × 10^−12^ F/m, and *ε*_*medium*_ is the dielectric constant of the medium. Re[*f*_*CM*_] is the real part of the complex Clausius-Mossotti factor, which describes the polarisation of the cell with respect to the surrounding medium. Re[*f*_*CM*_] is a function of the cell geometrical and dielectric properties, medium dielectric properties, and the frequency of the applied signal energising the microelectrodes, as detailed in Supplementary Information 1. Finally, *E*_*RMS*_ is the root-mean-square value of the electric field induced by microelectrodes.

To demonstrate the capabilities of our approach for the robust immobilisation of cells, we have presented results using a HEK-293 cell suspension. To calculate the Re[*f*_*CM*_] of HEK-293 cells, we determined the first crossover frequency of cells, where transition from negative dielectrophoresis (repelling from the microelectrodes) to positive dielectrophoresis (attracting to the microelectrodes) occurs. The crossover frequencies of cells measured at three medium electrical conductivities (*σ*_*medium*_) of 0.02, 0.05, 0.1 S/m, were obtained as 55 ± 7, 145 ± 17, and 285 ± 45 kHz, respectively. Using the crossover frequency of cells, the Re[*f*_*CM*_] of HEK-293 cells was calculated over the frequency range of 10^4^ to 10^8^ Hz (Fig. 1c). In our experiments, the electrical conductivity of the medium was set to 0.02 S/m. Immobilisation of the HEK-293 cells was achieved by applying a sinusoidal signal of 5 V_pk-pk_, at a frequency of 10 MHz, to maximise the magnitude of Re[*f*_*CM*_]; whilst, minimising the occurrence of electrolysis and electrothermal vortices, which occur at low frequencies.

Figure 1d presents the distribution of electric field generated over the glass substrate, obtained by numerical simulations, as detailed in Supplementary Information 2. Simulations reveal the generation of a strong electric field in the area between the microelectrodes. When applying a sinusoidal signal of 5 V_pk-pk_, electric field strength reaches a maximum of 126 kV/m along the edges of microelectrodes. The overlapped region of the microelectrodes lies along the middle of the microchannel, and enables the quick trapping of the moving cells. Figure 1e illustrates the contours of 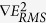, generated over the glass substrate, obtained by numerical simulations. According to Equation (1) this term is proportional to the DEP force experienced by the cells. Simulations indicated the generation of a strong DEP force along the edges of microelectrodes, with the maxima at the tip of microelectrodes. This enabled the immobilisation of cells along the entire length of microelectrodes. A stronger DEP force is induced at the tip of the microelectrodes, which potentially leads to the secondary layer of cells accumulating at the tips; however, this secondary layer is washed away upon increasing the flow rate, as described in the following section of the paper. This results in the formation of a single layer of cells immobilised along the width of the microfluidic channel.

### Cell immobilisation process

**Figure 2:**
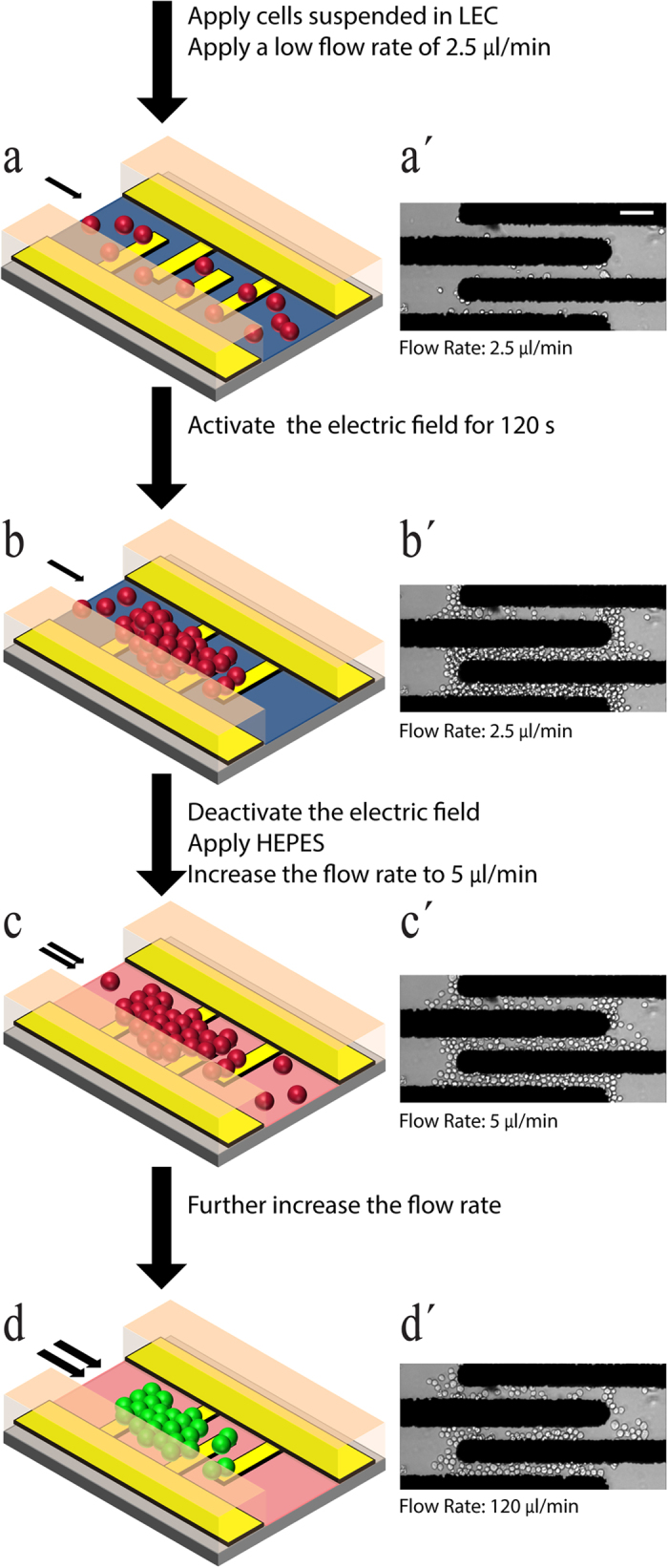
Robust Cell Immobilisation Process. (**a**–**d**) Schematic representation of the immobilisation process with (**a´**–**d´**) corresponding experimental results presented for the case when a high flow rate of 120 μl/min is applied to the immobilised cells. (**a**) Cells suspended in LEC are applied to the microfluidic platform at a low flow rate of 2.5 μl/min. (**b**) Electric field is activated for 120 s to immobilise the cells between the microelectrodes. (**c**) Electric field is deactivated, after which HEPES buffer is flushed through the microfluidic platform. After 10 min, the flow rate is increased to 5 μl/min to remove the loosely joint cells, and especially the secondary layers of cells. (**d**) The flow rate is then increased to desired values. Scale Bar: 50 μm.

The process used for robust immobilisation of cells is presented in Fig. 2. Initially, the glass substrate patterned with microelectrodes and the PDMS microchannel were cleaned with isopropanol and water, dried with nitrogen, and then assembled using a mechanical clamp to avoid leakage. In the case that the glass substrate and channel are not clean, the cells will not remain immobilised to the substrate in the presence of high flow rates. Water was then flushed through the microchannel, by applying a withdraw flow rate of 2.5 μl/min, and then the cells suspended in a LEC (comprising of 8.5% w/v sucrose and 0.3% dextrose in deionised water) buffer of 0.02 S/m were applied to the microchannel (Fig. 2a).

The microelectrodes were then activated using a 5 V_pk-pk_ AC sinusoid at 10 MHz, which led to the immobilisation of the cells. The electric field was kept active until the desired population of cells were patterned at the microelectrodes. For example, it takes 120 s to form the cell pattern presented in Fig. 2b.

The electric field was then deactivated and the cell suspension within the inlet reservoir was exchanged to HEPES, to ensure the viability and functioning of the cells. It should be noted that HEPES has an electrical conductivity of 1.2 S/m, such that it cannot be applied to the microchannel in the presence of electric field; if done so it leads to creation of strong electrothermal vortices and bubbles over the surface of microelectrodes. After a minimum incubation time of 10 min in HEPES buffer, which enabled cells to equilibrate, the flow rate was increased to 5 μl/min. The slight increase in flow rate facilitated the removal of loosely joint cells, and also the secondary layers of cells accumulated above the first layer of cells; in turn, leaving a clear view of the cells directly in contact with the substrate, upon observation with an inverted microscope (Fig. 2c). The flow rate was then increased, for example to 120 μl/min to enable the analysis of cells when stimulated with flow-induced shear stress (Fig. 2d).

At the completion of every experiment, the viability of the cells was examined on-chip, through staining the cells with propidium iodide (PI). PI was applied to the cells at a flow rate of 2.5 μl/min for 5 min, to ensure sufficient staining, and the response of cells was observed through the inverted microscope. A similar process was used for the stable immobilisation of *Saccharomyces cerevisiae* yeast cells.

### Cell trapping efficiency

To assess the capability of our approach for the stable immobilisation of cells, we measured the trapping efficiency of the HEK-293 cells when exposed to flow-induced shear stress. These cells are loosely adherent cells, and can be easily dislodged by fluid flow. For comparison, the adhesion force of HEK-293 cell is one order of magnitude less than that of HeLa cell, which is an adherent cell line^41^. The common approach to improve the adhesion of these cells is to pre-coat the glass surface with poly-L-lysin or collagen^42^. Other methods such as surface modification of glass substrates with positively charged polymers such as poly-ethyleneimine^42^, and modifying cell’s integrin expression profile^43^, have been also demonstrated to enhance the adhesion properties of these cells.

**Figure 3:**
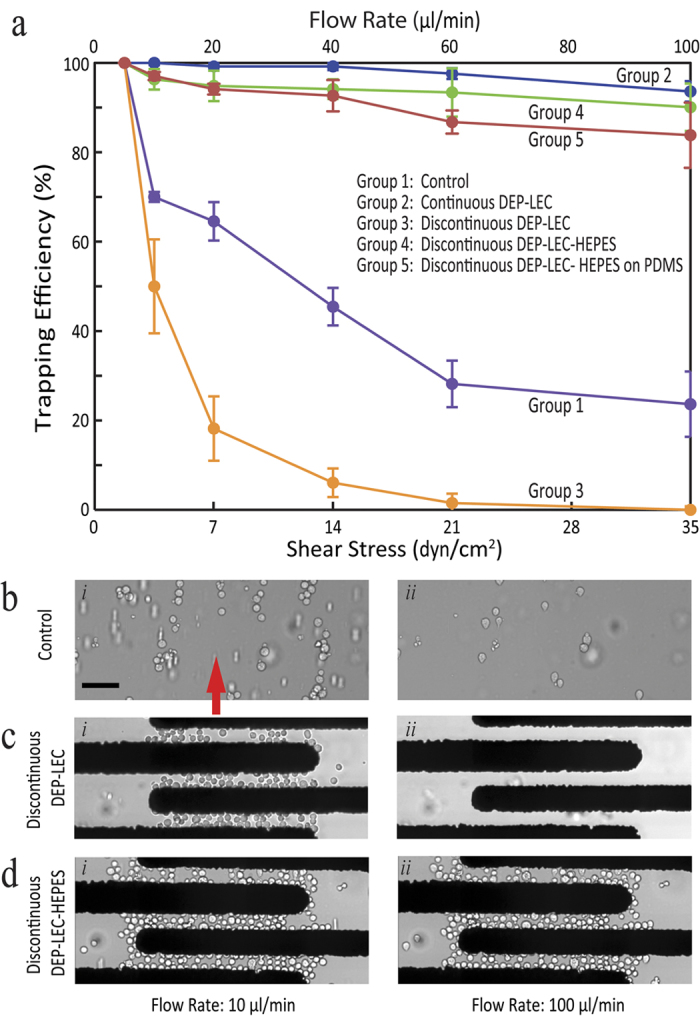
Cell Trapping Efficiency. (**a**) The trapping efficiency obtained for experimental groups one to five. (**b**–**d**) Images compare the cell population for experimental groups one, three and four, obtained at the flow rates of 10 and 100 μl/min. Group-one: no electric field is used to immobilise cells with cells suspended in HEPES, group-three: discontinuous DEP after 120 s with cells suspended in LEC, and group-four: discontinuous DEP after 120 s, after which the medium is changed to HEPES. The flow direction is indicated by the red arrow and applies to all presented experimental images. Data is representative of three to five independent experiments and error bars indicate mean ± SEM. Scale Bar: 50 μm.

In doing so, following the immobilisation of cells at a flow rate of 2.5 μl/min, the flow rate was sequentially increased every 3 min to 5, 10, 20, 40, 60, and 100 μl/min. The trapping efficiency was calculated as *ζ*_*trapping*_ *=* *n*_*remained*_*/n*_*initial*_ × 100%, in which *n* is the number of cells; with the initial value corresponding to the number of cells present at 5 μl/min, as presented in Fig. 3. Experiments were repeated three to five times.

Assuming that the patterned cells do not modify the hydrodynamics of the microfluidic channel, applying flow rates of 5, 10, 20, 40, 60, and 100 μl/min led to inducing of shear stress values of 1.75, 3.5, 7, 14, 21 and 35 dyn/cm^2^, respectively, over the surface of the cells. Shear stress values were obtained through numerical simulations, as detailed in Supplementary Information 3. For the microfluidic channel used in our experiments, the correlation between the flow rate and the shear stress can be expressed as *τ* = 67.2 *μ Q/ W H*^*2*^; where, *μ* is buffer’s viscosity, *Q* is flow rate, and *W* and *H* are the width and height of the microfluidic channel, respectively.

To determine the sensitivity of our cell immobilisation process to different operating conditions, we performed five groups of experiments; as explained below with the results presented in Fig. 3a:

- Group-one (control): no electric field was applied, and the cells suspended in HEPES were allowed to rest over the substrate within 30 min in the absence of flow (Fig. 3b);
- Group-two (continuous DEP-LEC): the electric field was applied through the entire duration of the experiment, and the cells were kept in LEC;
- Group-three (discontinuous DEP-LEC): the electric field was applied for only 120 s, and the cells were kept in LEC (Fig. 3c);
- Group-four (discontinuous DEP-LEC-HEPES): representing our immobilisation approach where the electric field was applied for only 120 s, after which the LEC suspension was exchanged for HEPES (Fig. 3d);
- Group-five (discontinuous DEP-LEC-HEPES on PDMS): similar to group-four, however the glass substrate was exchanged for a PDMS substrate.

It should be noted that in groups two to five, the electric field was generated by energising the microelectrodes with a 5 V_pk-pk_ AC sinusoid at 10 MHz. Group-one led to a trapping efficiency of 24%. The process of patterning cells was time-consuming and also did not offer any control over the location of patterned cells. In comparison, group-two led to a trapping efficiency of 93% at a flow rate of 100 μl/min, which was significantly higher than that of group-one (see Supplementary Movie 1). Despite such a high trapping efficiency, the continuous exposure of cells to electric field could potentially damage the cells^17^,^30^. In turn, this led us to discontinue the electric field after 120 s, which was long enough for a sufficient cell population to be immobilised (group-three). However, in this case, the trapping efficiency sharply reduced to 48% at a flow rate of 10 μl/min, and further dropped to zero at a flow rate of 100 μl/min (see Supplementary Movie 2). Such a weak trapping efficiency led us to exchange HEPES for LEC, after discontinuing the electric field (group-four). This led to a trapping efficiency of 91%, which was significantly higher than that of group-three, and was very similar to the results obtained from group-two (see Supplementary Movie 3). Our extended experiments indicated that for group-four experiments, the trapping efficiency is greater than 90%, even at a shear level of 63 dyn/cm^2^ (180 μl/min). However, at this shear stress level, some cells were seen to drift slightly, without detaching from the substrate.

The increased trapping efficiency obtained from group four, clearly highlights the role of cell extracellular environment in modulating the adhesion of cells to the glass substrate. This can be attributed to better adhesion properties of cell adhesion molecules (CAMs) in HEPES compared to LEC, enabling cells to withstand high shear stresses. CAMs are transmembrane proteins, which facilitate the adhesion of cells to a surface^44^. Among different CAMs, integrins are the major class of proteins involved in cell adhesion^44^,^45^. Several studies have revealed that the adhesion properties of integrins are strongly dependent on the presence and interplay of Ca^2+^and Mg^2+^cations in the buffer^46^,^47^. The absence of these two cations in LEC buffer can be the potential reason behind the decreased adhesion of HEK-293 cells to glass, in comparison to when the cells are suspended in HEPES.

To investigate if our immobilisation process depends on the material of the substrate, we replaced the glass substrate with a PDMS-coated substrate, with patterned gold/chrome microelectrodes on its surface^48^; where, we repeated the case of discontinuous DEP-LEC-HEPES on a PDMS substrate (group-five). This led to a trapping efficiency of 83% at a flow rate of 100 μl/min, which was slightly lower than that of group-four.

Furthermore, to investigate the effect of the applied voltage on the trapping efficiency of cells, we repeated the experiments associated with group-two, three, and four at different signal voltages of 2.5, 5, and 10 V_pk-pk_ (Supplementary Information 4). The results indicated that for the case of continuous DEP-LEC (group-two), increasing the voltage to 10 V_pk-pk_ reduces the trapping efficiency of cells. However, for the case of discontinuous DEP-LEC (group-three) changing the applied voltage did not improve the trapping efficiency. Alternatively, for the case of discontinuous DEP-LEC-HEPES (group-four), increasing the voltage to 10_pk-pk_ slightly improved the trapping efficiency at low flow rates of <20 μl/min but led to less trapping efficiencies at higher flow rates. These results suggest that an applied voltage of 5 V_pk-pk_ is optimal for the robust immobilisation of cells for group-four. Our experiments indicated that increasing the voltage escalates the density of cells immobilised between the first microelectrode pair, and leads to formation of multiple cells stacked on top of each other, which can be easily detached at high flow rates. Alternatively, at lower voltages (close to the optimal voltage of 5 V_pk-pk_) the cells were immobilised between the consequent microelectrode pairs more uniformly, and could withstand high flow rates. Moreover, applying high voltages might lead to conformational changes within the CAMs such as integrin, compromising their adhesion properties^49^.

In addition, in order to investigate the effect of the applied frequency on the trapping efficiency of cells, we repeated the group-four experiments at different frequencies ranging from 1 to 15 MHz. The results indicate that changing the frequency in the range of 5 to 12 MHz leads to similar trapping efficiencies obtained at 10 MHz.

Furthermore, we conducted a series of experiments to examine the proficiency of our approach for the robust immobilisation of *Saccharomyces cerevisiae* yeast cells. These cells have a poor adhesion to glass surfaces. Yeast cells are generally ellipsoidal (non-spherical) shaped, which further weakens their adhesion due to electrostatic and hydrophilic repulsive forces. Yeast cells that are more spherical in nature are more adhesive; additionally, their adhesion properties can be further improved by increasing the ionic strength of the buffer^50^.

The yeast cells were immobilised following the procedure shown in Fig. 2, and the trapping efficiency was measured across the flow rates of 10 to 100 μl/min (Supplementary Information 5). For the case of discontinuous DEP-LEC (group-three) a low trapping efficiency of 27% was obtained at a flow rate of 100 μl/min. In comparison, for the case of discontinuous DEP-LEC-HEPES, the trapping efficiency increased to 82% at the same flow rate (see Supplementary Movie 4). The increased trapping efficiency of yeasts in HEPES at high flow rates is in line with the results obtained for HEK-293 cells presented in Fig. 3a; and again highlights the role of cell extracellular environment in modulating the cell adhesion to the substrate. More importantly, it shows that our immobilisation approach is not limited to HEK-293 cells, and can potentially be applied to other non-adherent cells.

### Cell viability

To examine whether our immobilisation approach affects the viability of the cells, we compared the viability of the patterned cells over a 240 min duration, using propidium iodide (PI) staining and fluorescence microscopy. Following the immobilisation of cells at a flow rate of 2.5 μl/min, PI was flushed through the microchannel, and the viability was calculated as *ζ*_*viability*_ *=* *(n*_*total*_ − *n*_*PI positive*_*)/n*_*total*_ × 100%, where *n* is the number of cells.

**Figure 4:**
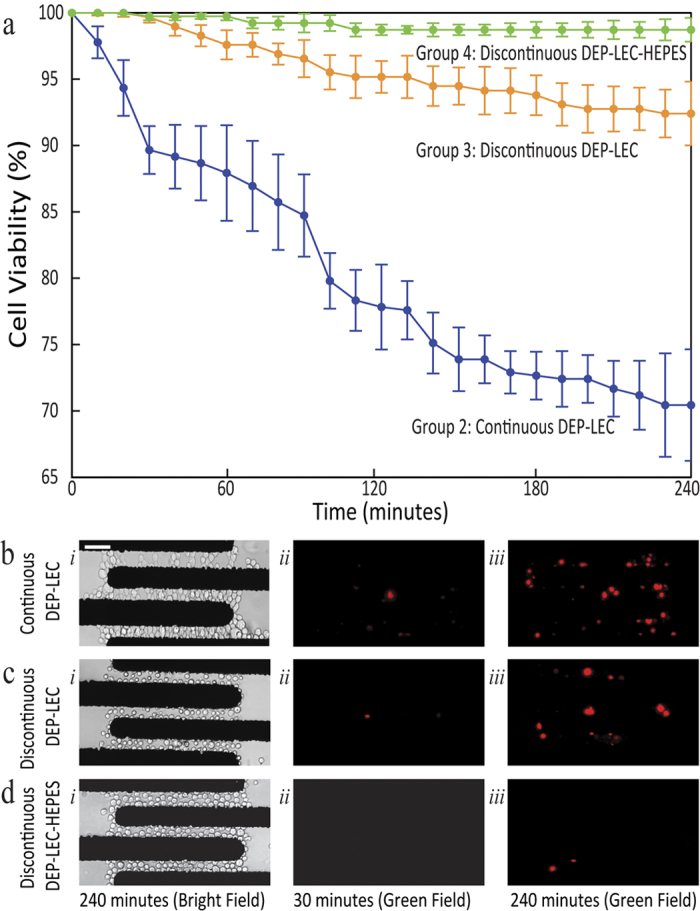
Viability of the Cells. (**a**) The viability of immobilised cells is displayed for three different groups, including group-two: continuous DEP-LEC, group-three: discontinuous DEP-LEC, and group-four: discontinuous DEP-LEC-HEPES. In all cases, fresh suspension medium either, LEC or HEPES was supplied continuously over 240 min, at a low flow rate of 2.5 μl/min. (**b**–**d**) Experimental results for each experimental group. (i) Bright field image, after 240 min, presenting the entire population of both viable and non-viable cells. (ii) Fluorescence images, highlighting the non-viable cells after 30 min. (iii) Fluorescence images, highlighting the non-viable cells after 240 min. Data is representative of three to three independent experiments and error bars indicate mean ± SEM. Scale Bar: 50 μm.

To identify the sensitivity of cells to operating conditions of the system, we performed three groups of experiments corresponding to group-two (continuous DEP-LEC), three (discontinuous DEP-LEC), and four (discontinuous DEP-LEC-HEPES), explained in the previous section, with the results presented in Fig. 4a.

Group-two (continuous DEP-LEC), which is commonly used in dielectrophoresis based experiments, led to a viability rate of 72% after 240 min (Fig. 4b). In comparison, group-three (discontinuous DEP-LEC) led to a viability rate of 92% after 240 min (Fig. 4c), which is significantly higher than that of group-two, and clearly indicates the adverse effect of continuous electric field on the viability of cells, which can be associated with the effects such as Joule heating of the surrounding medium, trans-membrane potential interaction and reorganisation/activation of cell membrane components^17^,^29^. Finally, group-four (discontinuous DEP-LEC-HEPES) led to a viability rate of 98% after 240 min (Fig. 4d), which is slightly higher than that of group-three, and highlights the adverse effect of LEC buffers on the viability of cells, which can be associated with the lack of various essential salts, the inability for maintaining physiological pH of the media, and insufficient conductivity similar to biological environments^16^,^51^.

### Shear-induced calcium signalling of TRPV4-HEK-293 cells

Due to the ability of the immobilised cells to withstand high flow rates, we were able to investigate the shear-induced calcium signalling of HEK-293 cells, stably expressing TRPV4 in a microfluidic channel; and compare them with the response of non-transfect HEK-293 cells.

**Figure 5:**
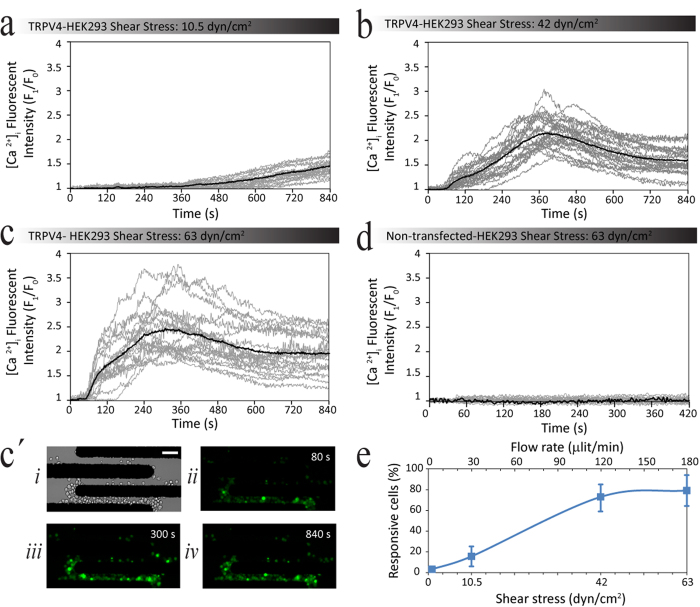
Application of the dielectrophoresis-based immobilisation process for analysis of shear-induced calcium signalling. (**a**–**d**) Each graph represents the normalised single cell intensity profiles of 30–50 cells obtained over a duration of 840 s under different shear stresses. Note that increasing the shear stress elevates the average fluorescent intensity from (**a**) 1.54 at 10 dyn/cm^2^ to (**b**) 2.27 and to (**c**) 2.49 at 42 and 63 dyn/cm^2^, respectively. (**c´**) shows representative (i) bright field and (ii-iv) fluorescent images of immobilised TRPV4-HEK-293 cells loaded with Fluo-4AM acquired at 80, 300, and 840 s upon application of 63 dyn/cm^2^ shear stress. (**d**) non-transfected HEK-293 cells did not show any response to shear stress even at 63 dyn/cm^2^. (**e**) Bar graph shows that increasing the shear stress elevates the percentage of activated cells. Data is representative of three independent experiments and error bars indicate mean ± SEM. Note that an increase in Fluo-4AM fluorescent intensity indicates increase in [Ca^2+^]_i_. Scale Bar: 50 μm.

Cells were loaded for 30 min with Fluo-4AM dye as previously described^38^, and then carefully diluted in LEC, such that 20 μl of the loaded cells were suspended into 1000 μl of LEC; ensuring to avoid pre-exposure of cells to shear stress. Once the cells were immobilised by means of dielectrophoresis (explained in Fig. 1), the flow rate was increased to achieve the desired level of shear stress. Simultaneously, the intracellular calcium level, [Ca^2+^]_i_ was quantified by measuring the changes in the intensity of the Fluo-4AM compared to the basal level, as presented in Fig. 5.

The Fluo-4AM fluorescent intensity measurements obtained over a duration of 840 s were normalised to the resting cell intensity, to obtain an intensity profile for individual cells at different flow rates of 30 μl/min (Fig. 5a), 120 μl/min (Fig. 5b), and 180 μl/min (Fig. 5c). Three time-lapse images taken at 80, 300, and 840 s are presented in Fig. 5c´, respectively for a flow rate of 180 μl/min. Time-lapse images for flow rates of 30, and 20 μl/min are given in Supplementary Information 6.

Our experiments indicated that higher levels of shear stress reduce the time taken for cellular calcium levels to start increasing. For example, at shear stress values of 42 dyn/cm^2^ (120 μl/min) and 63 dyn/cm^2^ (180 μl/min), TRPV4-HEK-293 cells responded to shear within 77 ± 6 and 53 ± 21 s, respectively; whilst, at a shear stress of 10.5 dyn/cm^2^ (30 μl/min), the cellular calcium was still increasing slowly at 360 ± 28 s, and did not reach a plateau during the duration of the experiment. In addition, experiments indicated that higher levels of shear stress reduce the time taken for cellular calcium levels to reach their maximum value. For example, increasing the shear to 42 dyn/cm^2^ (120 μl/min) and 63 dyn/cm^2^ (180 μl/min), reduced the peak response time to 392 ± 18 and 253 ± 32 s, respectively (Fig. 5a–c).

Furthermore, we found that increasing the shear stress increases the magnitude of the maximal intracellular calcium level from 1.46 ± 0.03 at 10.5 dyn/cm^2^ (30 μl/min), to 2.27 ± 0.07 and 2.49 ± 0.1 at 42 dyn/cm^2^ (120 μl/min), and 63 dyn/cm^2^ (180 μl/min), respectively (Fig. 5a–c). It should be noted that the control negative group (non-transfected HEK-293 cells) did not show any response to shear stress (Fig. 5d). Additionally, our experiments indicated that increasing the shear stress elevates the percentage of activated cells from 3.25 ± 1.2% at a shear level of 1.75 dyn/cm^2^ to 15.67 ± 8.9, 73.1 ± 12.5, and 79.3 ± 14.3, at shear levels of 10.5, 42 and 63 dyn/cm^2^, respectively (Fig. 5e).

At the completion of the experiment, the viability of the cells was examined by applying PI suspended in HEPES, at a flow rate of 2.5 μl/min. The calcium signalling of the non-viable cells was excluded from the analysis.

## Discussion

Here, we have demonstrated the robust immobilisation of cells using discontinuous dielectrophoresis. In contrast to conventional immobilisation methods, our approach does not rely on substrate modification. The method enables dynamic control of the microenvironment, and parallelisation. More importantly, it provides the ability to apply a very high magnitude of shear over the immobilised cells, which was not previously possible.

The proposed approach will open new horizons to study the mechanotransduction of non-adherent cells. Mechanotransduction is a process by which cells are able to sense the mechanical stress that they receive from the surrounding environment, and translate it into biochemical signals. Cellular response to mechanical stress modulates a diverse range of cellular functions such as proliferation, differentiation, migration, and is crucial for cellular homeostasis and function^52^.

Due to limitation of available tools, the identity and mechanism of action of mammalian mechanoreceptors remains poorly understood. It is not clear how cells transduce the mechanical signals that they receive from the surrounding environment; and there is much debate about the nature of the primary mechanosensing molecules. Mechanical stimulation across the full physiological range remains difficult for non-adherent or loosely adherent cells, since they might be washed away using conventional approaches.

The proposed method allows us to stably immobilise cells in suspension, and apply shear stress from the low end of the physiological range to the high end; whilst, measuring fluorescent readouts of individual cellular responses using microscopy. Immobilisation was achieved by activating the electric field for a short duration of 120 s, after which the electric field was deactivated and HEPES was flushed through the microchannel. This approach overcomes two major limitations of dielectrophoresis, including the long-term exposure to strong electric fields, and the use of low electrical conductivity isotonic buffers, which both can affect the viability and functionality of cells. It should be noted that the use of dielectrophoresis to immobilise different mammalian cells while maintaining their viability and functionality has been demonstrated in several studies^30^,^34^,^53^,^54^,^55^. The magnitude of electric field, and the time that immobilised cells are exposed to electric field, are the two major factors that can contribute to the loss of viability and functionality of cells. To highlight the features of our approach, we have compared these two factors in our study to conditions used in the literature, as given in Table 1. The maximum electric field applied to the cells in our study is 126 kV/m (Fig. 2d), which is comparable to values in the literature; however, the time that cells are exposed is limited to 120 s in our study, which is considerably less than those in the literature, which is less likely to damage fragile mammalian cells.

We are interested in mechanosensitive ion channels and changes of [Ca^2+^]_i_ and the majority of available literature on shear stress studies is limited to low shear flows. The ability to examine high shear flows is important, as there is a potential for other mechanosensitive ion channels that are yet to be identified due to possessing a high shear threshold.

Using this method, the shear stress response of TRPV4 channels was investigated. TRPV4 is a Ca^2+^ permeable ion channel, wildly expressed in different organs; additionally, its response to mechanical stress is linked to different physiological and pathological conditions^36^,^38^,^56^. For example, in vascular endothelium TRPV4 controls the vasodilation of smooth muscle cells in response shear stress^56^. As such, understanding the signalling pathways regulating mechanosensitivity of TRPV4 is highly valuable for clinical pharmacology. So far available literature has not been able to report a clear shear stress response from cells expressing TRPV4; which will influence understanding of underlying subcellular responses, mainly due to limitation of available methods. Using our approach, we were able to apply high levels of shear stress, and obtained clear increase in [Ca^2+^]_i_ that was dose dependent, and also influenced the percentage of cells responding to shear stress.

Although we used our approach for studying the shear-induced calcium signalling of cells, it has the potential to be applied to a wide range of cellular assays, in particular, where the non-adherent cells are required to be immobilised, then stimulated either chemically or physically, and monitored in real-time.

## Methods

### Fabrication of the microelectrodes

For the microelectrodes, gold on chrome films were deposited using physical vapour deposition onto a glass substrate at thickness of 1500 Å and 500 Å, respectively. The microelectrodes were then patterned using standard microfabrication techniques including photolithography and etching.

### Fabrication of the microchannel

The microchannel was fabricated utilising soft lithography and replica molding techniques^57^. The polydimethysiloxane (PDMS) was prepared using Sylgard 184, with the base to curing agent ratio 10:1 (Dow Corning Corporation, MI). A PDMS microchannel of 500 × 80 μm (W × H) was used to apply appropriate suspensions, including the cells in the vicinity of the microelectrodes.

### Cell preparation

HEK-293 T-REx (Life Sciences) cell lines stably expressing human TRPV4 were generated as reported elsewhere^58^. Cells were grown in tetracycline-free DMEM media supplemented 10% FBS, blasticidin (5 μg/ml) and hygromycin (50 μg/ml). TRP channel expression was induced using 0.1 μg/ml of tetracycline 12 h before each experiment. As negative control we used HEK-293 T-REx cells that were not transfected.

Changes in intracellular [Ca^2+^]_i_ were measured on cell suspensions obtained by trypsinization of confluent monolayers of wild-type and TRPV4 stably transfected HEK-293 cells. The suspended cells were loaded with Fluo-4AM, washed twice with HEPES-buffered saline solution (140 mM NaCl, 5 mM KCl, 10 mM HEPES, 11 mM D-glucose, 1 mM MgCl_2_, 2 mM CaCl_2_, and 2 mM probenecid, adjusted to pH 7.4), and assayed for intracellular calcium concentration ([Ca^2+^]_i_). Intensity measurements were obtained using an inverted microscope (Nikon Eclipse, TE 2000) equipped with a photomultiplier tube, a near infrared camera (QuantEM:512SC, Photometrics), and a 10× objective (CFI Plan Apo Lambda 10×). The intensity measurements were then processed using NIS Elements, microscope imaging software (Basic Research, Nikon Instruments), thus, enabling the intensity profiles to be obtained.

At the completion of every experiment, the viability of the cells was examined on-chip with propidium iodide (PI) (10 μg/ml) staining.

## Additional Information

**How to cite this article**: Soffe, R. *et al.* Analysing calcium signalling of cells under high shear flows using discontinuous dielectrophoresis. *Sci. Rep.* **5**, 11973; doi: 10.1038/srep11973 (2015).

## Supplementary Material

Supplementary Information

Supplementary Movie 1

Supplementary Movie 2

Supplementary Movie 3

Supplementary Movie 4

## Figures and Tables

**Table 1:** Comparing the magnitude of electric field and duration of exposure to electric field in our study to the ones in the literature.

Cell type	Purpose	Magnitude of electric field	Duration of exposure to electric field	Ref.
U937 Leukemia	Monitoring the response of cells to apoptosis inducer drugs	>250 kV/m	210 min	30
Jurkat T-cells	Making 3D aggregation of cells for tissue engineering studies	>150 kV/m	30 min	34
SAOS-2 Osteoblasts				
A549-luc-C8 Human lung cancer	Separation and collection of viable lung cancer cells, followed by extraction of the nuclei of cells	>150 kV/m	~15 min	53
CD34 + Stem cell	Isolating CD34 + cells from bone marrow and peripheral blood samples	>75 kV/m	10 min	54
MDA231 Human metastatic breast cancer	Separation of breast cancer cells from blood	>60 kV/m	10 min	55
**Our approach**	**Robust immobilisation of cells to analyse their shear-induced calcium signalling**	**126** **kV/m**	**2** **min**	**Current**
