# Supplementary material for: Analysing calcium signalling of cells under high shear flows using discontinuous dielectrophoresis
Source: Sci Rep. 2015 Jul 23;5:11973. doi: 10.1038/srep11973 (PMC4648442; doi:10.1038/srep11973)
Supplement: Supplementary Information [file srep11973-s1.pdf]

Supplementary Information:

Analysing calcium signalling of cells under high shear flows using discontinuous dielectrophoresis

Rebecca Soffe, [1] Sara Baratchi, [2] Shi-Yang Tang, [1] Mahyar Nasabi, [1]
Peter McIntyre, [2] Arnan Mitchell, [1] Khashayar Khoshmanesh [1,*]

[1] School of Electrical and Computer Engineering, RMIT University, Melbourne, Australia

[2] Health Innovations Research Institute and School of Medical Sciences, RMIT University, Melbourne, Australia

Corresponding Author:

khashayar.khoshmanesh@rmit.edu.au

Supplementary Information S1: Determining the Clausius-Mossotti factor for HEK-293 cells

Considering the HEK-293 cells as a homogenous spherical structure, the Clausius-Mosotti factor, f_{CM} can be evaluated as follows:[1]

$$f_{CM} = \frac{\varepsilon^*_{cell} - \varepsilon^*_{medium}}{\varepsilon^*_{cell} + 2\varepsilon^*_{medium}} \tag{S1}$$

$$\varepsilon^* = \varepsilon - \frac{i\sigma}{\omega}, \; i = \sqrt{-1}, \tag{S2}$$

where, ε^* and ε are the complex and real permittivity, respectively, σ is the electrical conductivity, and ω is the angular frequency of the applied alternating-current signal.

HEK-293 cells have a single layer structure, thus, the single-shell spherical model is applied to predict f_{CM}. The equivalent complex permittivity of the cell, encompassing the cell membrane and cytoplasm is determined by:[2]

$$\varepsilon^*_{cell} = \varepsilon^*_{membrane} \frac{\left[\frac{r_{membrane}}{r_{cytoplasm}}\right]^3 + 2\left[\frac{\varepsilon^*_{cytoplasm} - \varepsilon^*_{membrane}}{\varepsilon^*_{cytoplasm} + 2\varepsilon^*_{membrane}}\right]}{\left[\frac{r_{membrane}}{r_{cytoplasm}}\right]^3 - \left[\frac{\varepsilon^*_{cytoplasm} - \varepsilon^*_{membrane}}{\varepsilon^*_{cytoplasm} + 2\varepsilon^*_{membrane}}\right]} \tag{S3}$$

The geometric and dielectric properties of HEK-293 cells are given in Supplementary **Table S1**.

Table S1. Geometric and dielectric properties of HEK-293 cells (ε_0=8.854×10^{-12} F/m)

Properties	Viable Cells
r (μm)	8
$\sigma_{cytoplasm}$ (S/m)	0.5
$\varepsilon_{cytoplasm}$ (F/m)	60 ε_0
$\sigma_{membrane}$ (S/m)	7×10^{-14}
$\varepsilon_{membrane}$ (F/m)	9.5 ε_0
$t_{membrane}$ (nm)	7

Supplementary Information S2: Calculation of electric field and dielectrophoretic (DEP) force induced by microelectrodes

In order to calculate the contours of electric field, the Laplace equation was solved within the microfluidic channel by applying appropriate electric potentials at the microelectrodes while zero electric flux at other surfaces of the chamber, including the bottom, top and sidewalls:

$$\nabla^2 \phi_{rms} = 0 \qquad \text{(S4)}$$

The electric field was calculated by differentiating the electric potential:

$$E = -\nabla \phi_{rms} \qquad \text{(S5)}$$

The DEP force was calculated by calculating the gradient of electric field square:

$$F_{DEP} \propto \nabla E^2 \qquad \text{(S6)}$$

Figure S1 illustrates the contours of E and ∇E^2 produced by inter-digital microelectrodes used for robust immobilisation of cells.

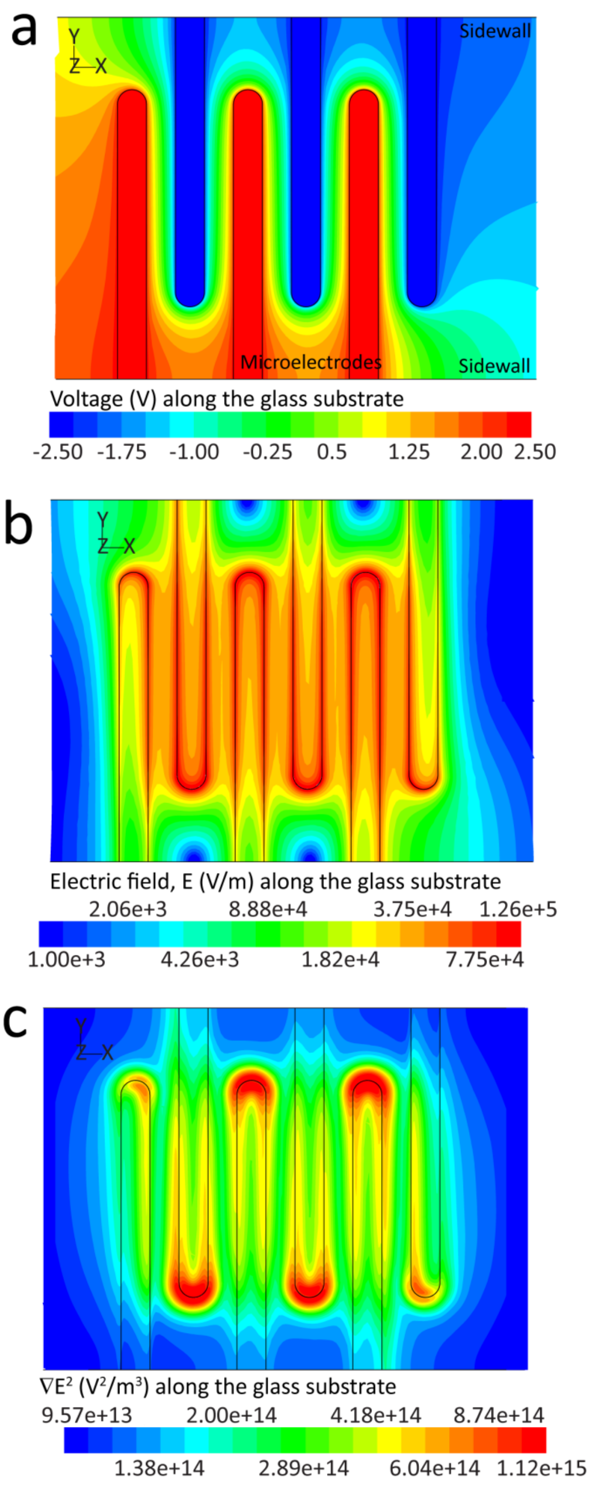

Figure S1. Characterisation of microelectrodes used for robust immobilisation of cells. (a) Contours of electric potential, φ (b) Contours of electric field, E (c) Contours of the gradient of electric field square, ∇E^2 obtained by numerical simulations.

Supplementary Information S3: Velocity and shear stress profiles

Computational Fluid Dynamics (CFD) technique was used to calculate the variations of shear stress at the glass substrate. Considering that flow is laminar and assuming that the liquid is Newtonian, the differential equations corresponding to the balance of mass (continuity) and momentum of the liquid as given below:

$$\nabla \cdot \vec{U} = 0 \tag{S7}$$

$$\rho_{liquid}(\vec{U} \cdot \nabla)\vec{U} = -\nabla P + \mu_{liquid} \nabla^2 \vec{U} \tag{S8}$$

in which $\vec{U}$, P are the velocity and pressure of the liquid, while ρ, μ, are the density and dynamic viscosity of the liquid, respectively. The boundary conditions include ambient pressure at the inlet, desired flow rates at the outlet, and no-slip at the walls.

Figure S2 illustrates the distribution of velocity along the cross section of the microchannel obtained by CFD simulations.

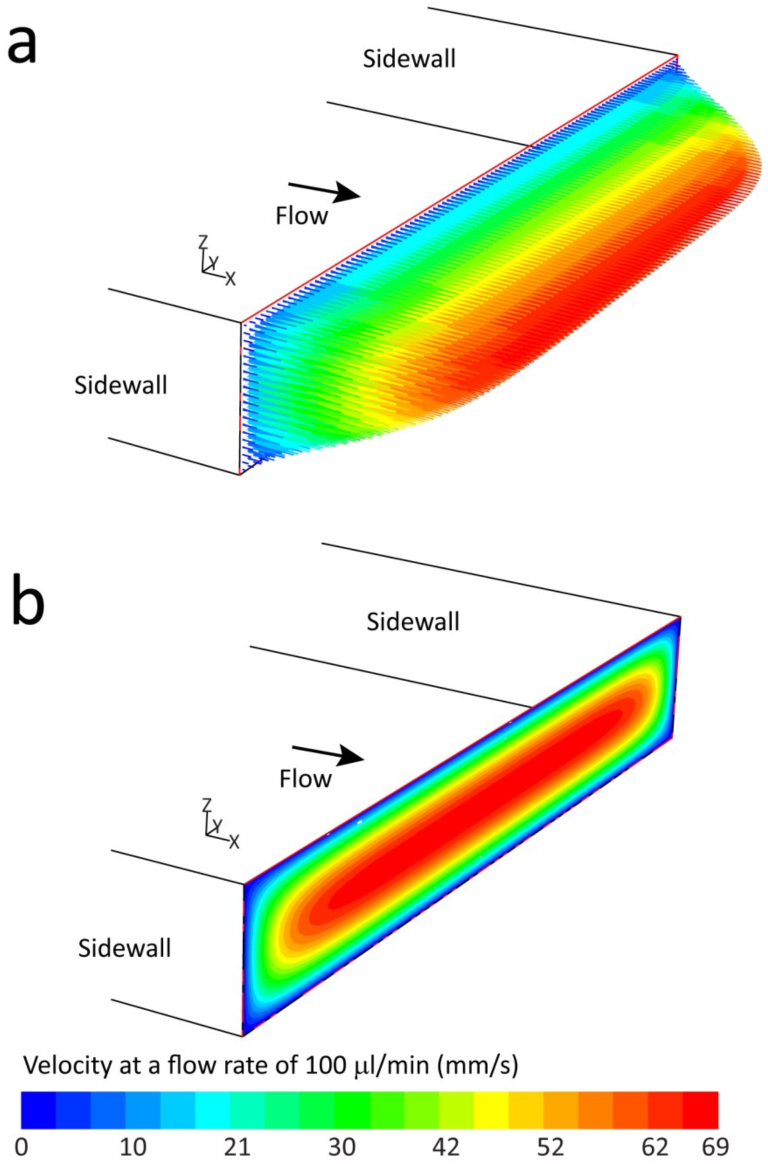

Figure S2. Distribution of velocity along the cross section of the microchannel. (a) Velocity vectors, (b) Velocity contours obtained by computational fluid dynamics (CFD) simulations.

Assuming that the immobilised cells do not alter the flow field, the shear stress applied over the glass substrate is obtained, as below:

$$\tau\Big|_{glasssubstrate} = \mu_{liquid} \frac{\partial U}{\partial z}\Big|_{z=0} \quad \text{(S9)}$$

Figure S3 illustrates the distribution of shear stress over the glass substrate, obtained by CFD simulations.

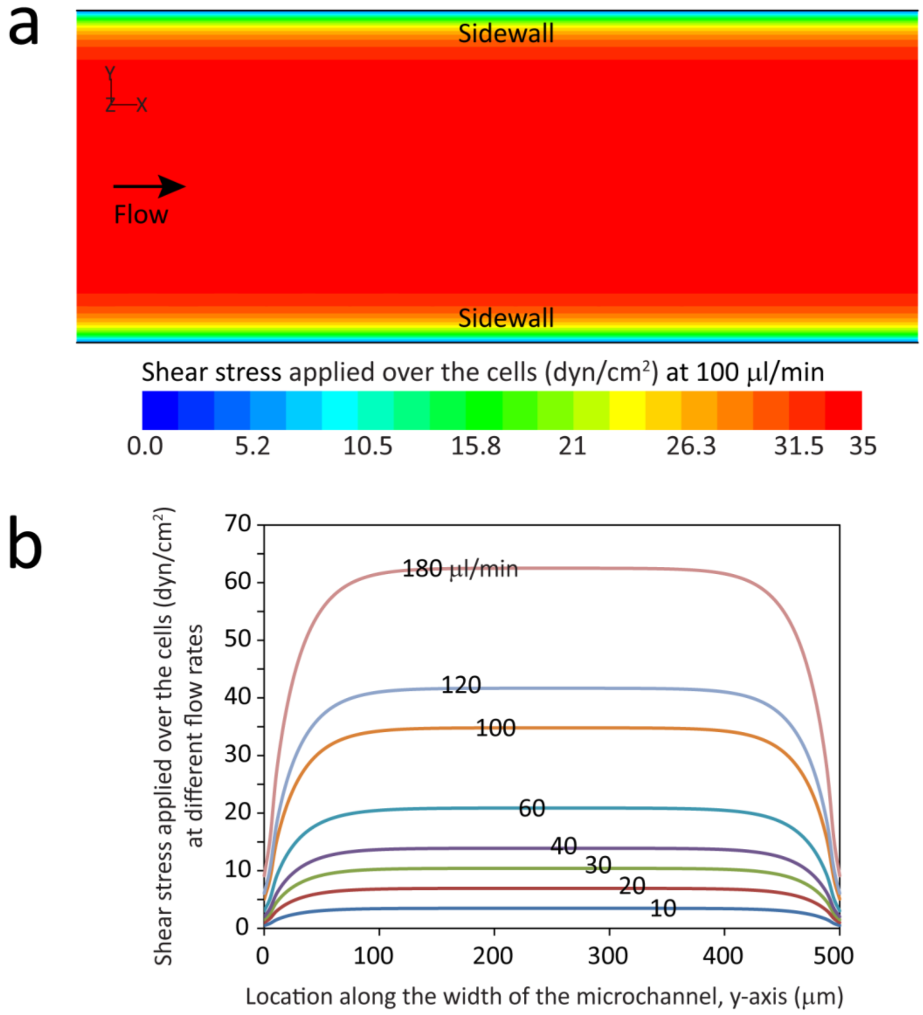

Figure S3. Variations of shear stress over the bottom surface of the microchannel. (a) Contours of shear stress obtained at a flow rate of 100 µl/min. (b) variations of shear stress applied over cells across the width of the microchannel at different flow rates of 10, 20, 30, 40, 60, 100, 120 and 180 µl/min.

Supplementary Information S4: Characterisation of trapping efficiency of HEK-293 cells at different signal voltages

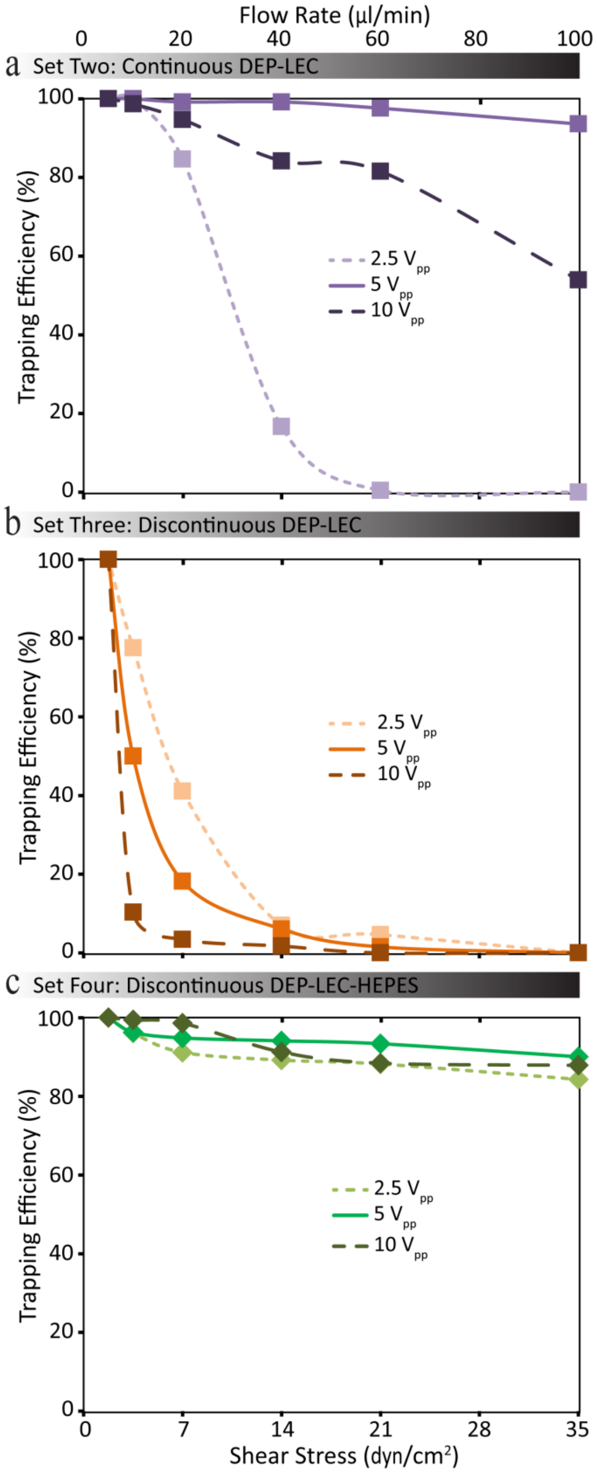

Figure S4. Characterising the trapping efficiency of HEK-293 cells at different operating voltages of 2.5, 5 and 10 $V_{p\text{-}p}$ at different operating conditions. (a) Group two: Continuous DEP-LEC. (b) Group three: discontinuous DEP-LEC. (c) Set four: discontinuous DEP-LEC-HEPES.

Supplementary Information S5: Robust immobilisation of yeast cells using our dielectrophoresis enabled immobilisation approach for the

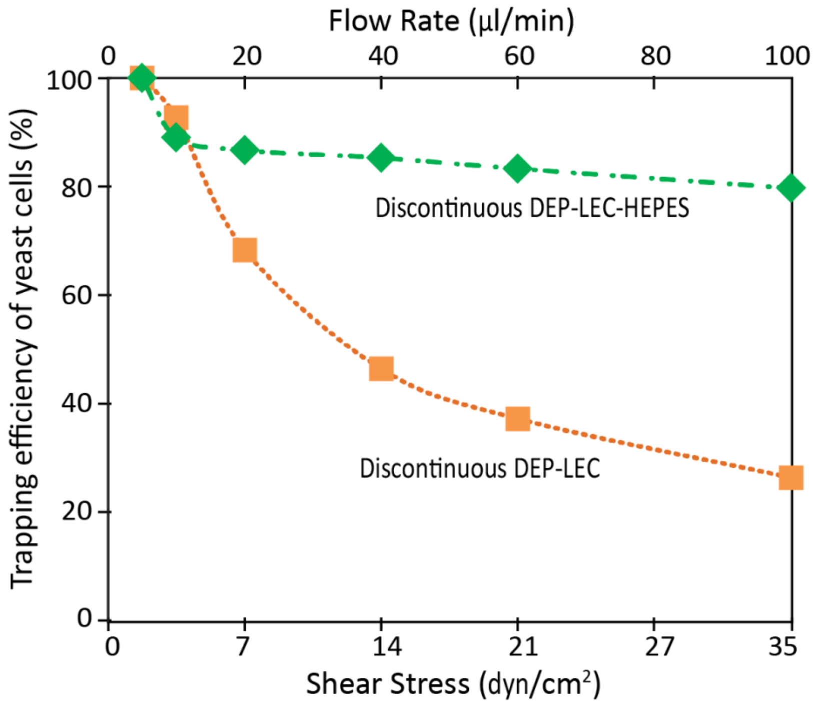

Figure S5. Trapping efficiency of yeast cells in two operating conditions: Discontinuous DEP-LEC (similar to Group-three presented in **Figure 3a**) and Discontinuous DEP-LEC-HEPES (similar to Group-four presented in **Figure 3a**).

Supplementary Information S6: Shear-induced calcium signalling of immobilised HEK-293 cells

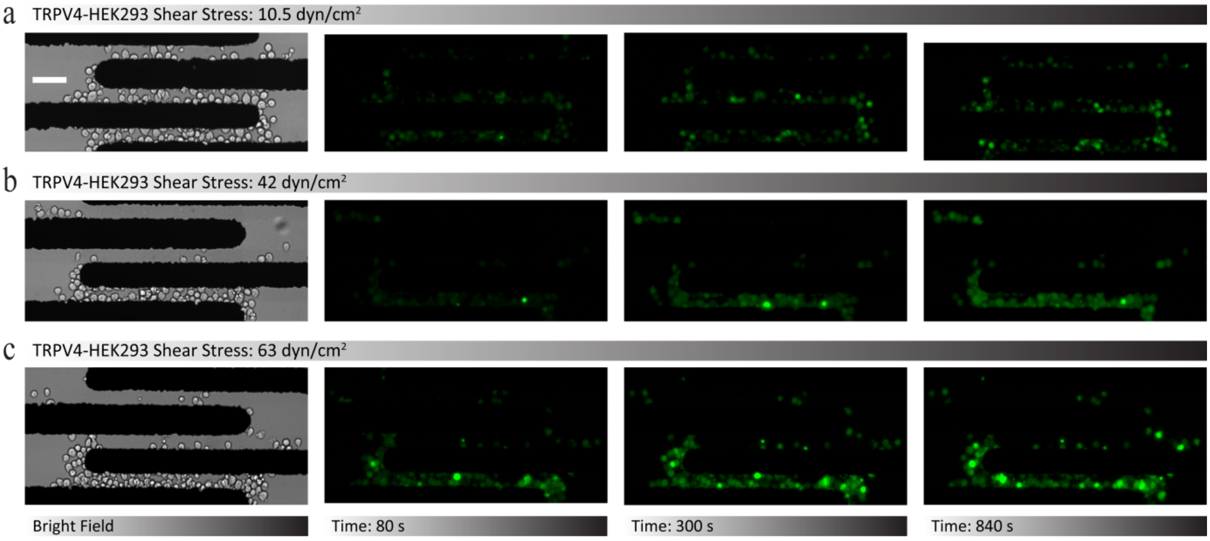

Figure S6. Shows representative bright field and fluorescent images of immobilised TRPV4-HEK293 cells loaded with Fluo-4AM acquired at 80, 300, and 840 s upon application of (a) 10.5 dyn/cm^2 shear stress corresponding to a flow rate of 30 µl/min. (b) 42 dyn/cm^2 shear stress corresponding to a flow rate of 120 µl/min. (c) 63 dyn/cm^2 shear stress corresponding to a flow rate of 180 µl/min.

References

1 Morgan, H. & Green, N. Research Studies Press Ltd., Baldock, England, 2003.

2 Huang, Y., Holzel, R., Pethig, R. & Xiao, B. W. Differences in the AC electrodynamics of viable and non-viable yeast cells determined through combined dielectrophoresis and electrorotation studies. *Physics in Medicine and Biology* **37**, 1499 (1992).
